# Role of Multislice Computed Tomography and Local Contrast in the Diagnosis and Characterization of Choanal Atresia

**DOI:** 10.1155/2011/280763

**Published:** 2011-05-22

**Authors:** Khaled Al-Noury, Alsaid Lotfy

**Affiliations:** ^1^Department of Otolaryngology, King Abdulaziz University, P.O. Box 35135, Jeddah 21488, Saudi Arabia; ^2^Radiology Department, International Medical Centre, P.O. Box 2172, Jeddah 21451, Saudi Arabia

## Abstract

*Objective*. To illustrate the role of multislice computed tomography and local contrast instillation in the diagnosis and characterization of choanal atresia. To review the common associated radiological findings. *Methods*. We analyzed 9 pediatric patients (5 males and 4 females) with suspected choanal atresia by multislice computed tomography. We recorded the type of atresia plate and other congenital malformations of the skull. *Results*. Multislice computed tomography with local contrast installed delineated the posterior choanae. Three patients had unilateral mixed membranous and bony atresia. Three patients had unilateral pure bony atresia. Only 1 of 7 patients have bilateral bony atresia. It also showed other congenital anomalies in the head region. One patient is with an ear abnormality. One patient had congenital nasal pyriform aperture stenosis. One of these patients had several congenital abnormalities, including cardiac and renal deformities and a hypoplastic lateral semicircular canal. Of the 6 patients diagnosed to have choanal atresia, 1 patient had esophageal atresia and a tracheoesophageal fistula. The remaining patients had no other CHARGE syndrome lesions. *Conclusions*. Local Contrast medium with the application of the low-dose technique helps to delineate the cause of the nasal obstruction avoiding a high radiation dose to the child.

## 1. Introduction

Choanal atresia is a congenital anomaly usually diagnosed at birth. It results from the developmental failure of the posterior nasal cavity (choanae) to communicate with the nasopharynx [1]. It is postulated to be secondary to an abnormality during the rupture of the buccopharyngeal membrane in the embryological period [2]. Choanal atresia is the most common form of congenital nasal obstruction [3]. Obstruction of the nasal airway may also result from turbinate hypertrophy, pyriform aperture stenosis, and nasal cavity stenosis in association with craniofacial anomalies [2]. 

The anatomic classification of choanal atresia has been revised [4]. It was previously thought that choanal atresia was bony in 90% of cases and membranous in 10% of cases [5]; however, the development of more accurate evaluation techniques indicated that most, if not all, patients with choanal atresia have bony abnormalities. 

**Figure 1:**
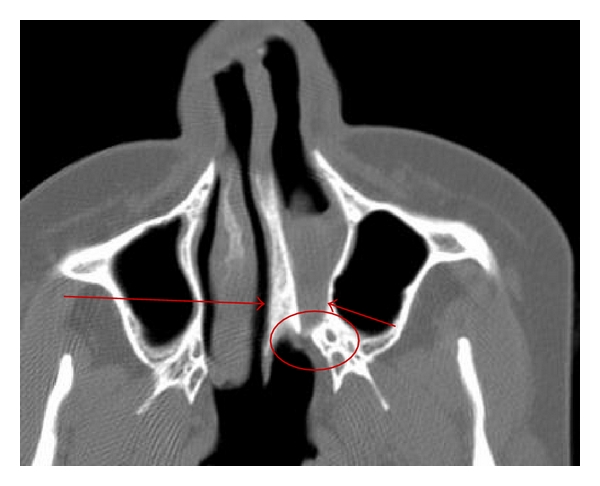
Axial Ct widening of the vomer (long arrow), bowing of the posteromedial maxilla (short arrow), and narrowing of the choana anterior to the pterygoid.

The major bony component of atresia is an abnormal widening of the vomer [6]. The posteromedial maxilla is bowed medially and approximates, or is fused with, the lateral margin of the vomer (Figure 1) [6]. The membranous components of atresia are characterized by soft tissue filling the posterior choanae associated with a narrowing of the posterior choanae just anterior to the pterygoid plates (Figure 1) [6].

The obstructing choanal membrane may be thin and strandlike or thick and pluglike. It is considered that combined bony and membranous malformations are found in approximately 70% of cases and purely bony atresia in approximately 30% of cases. The existence of pure membranous atresia without a bony abnormality has been disputed. 

Bilateral choanal atresia is an otolaryngology emergency. It is a potentially life-threatening disorder because the affected infant is an obligate nose breather [2]. Symptoms range from intermittent to severe respiratory distress with cyanosis that is aggravated by feeding and alleviated by crying. Approximately, 75% of children with bilateral choanal atresia have other congenital abnormalities, as exemplified by CHARGE syndrome (coloboma of eye or microphthalmia, heart malformation, choanal atresia, retarded growth, genital hypoplasia, and ear abnormalities, typically external) [7]. 

Surgical correction of bilateral choanal atresia is performed as soon as possible after the diagnosis has been made; however, there is little consensus regarding the optimal surgical approach [8]. Transpalatal, transnasal, transseptal, and sublabial approaches have been utilized with varying degrees of success [8]. Recently, the transnasal endoscopic procedure has been advocated as a safe and efficacious method with the best possibility for long-term nasal patency [8, 9]. 

The surgical approach is determined by the thickness of the bony atresia plate as determined by computed tomography (CT) [10]. CT scanning is often used as a diagnostic tool for choanal atresia because it can generate information regarding the extent and type of atresia [1]. 

The objective of this study was to illustrate the role of multislice CT and local contrast instillation for the diagnosis and characterization of choanal atresia as well as to highlight other associated congenital anomalies.

## 2. Methods

Following the approval from the hospital ethics committee, newborn patients with suspected choanal atresia due to symptoms of respiratory distress with cyanosis that is aggravated by feeding and alleviated by crying or suspected during nasal suction by attending pediatrician. 9 patients (5 males and 4 females) were included in this study between January 2007 and March 2010. All patients were examined by multislice CT using a GE high-speed 8-slice machine. 

The patients were positioned in the supine position with their head positioned to make their face perpendicular to the gantry (chin raised); the head was supported by a positioning pad. Patients were sedated using chloral hydrate. Nasal suction was performed and topical nasal vasoconstriction was installed just prior to examination the images were reformatted in different planes: axial (so that the posterior choanae were at the same level as the nasopharynx), coronal, and sagittal views. 

## 3. Results

The posterior choanae and their communication with the nasopharynx were clearly delineated in all patients (Table 1).

**Figure 2:**
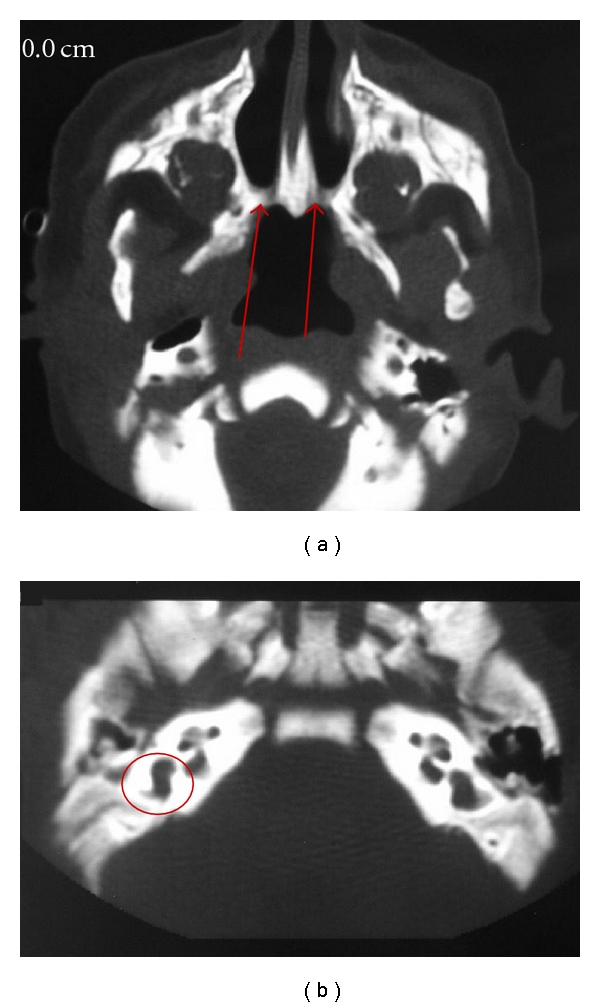
Bilateral bony atresia (a) (arrow), with internal ear abnormalities (b) (circle).

**Figure 3:**
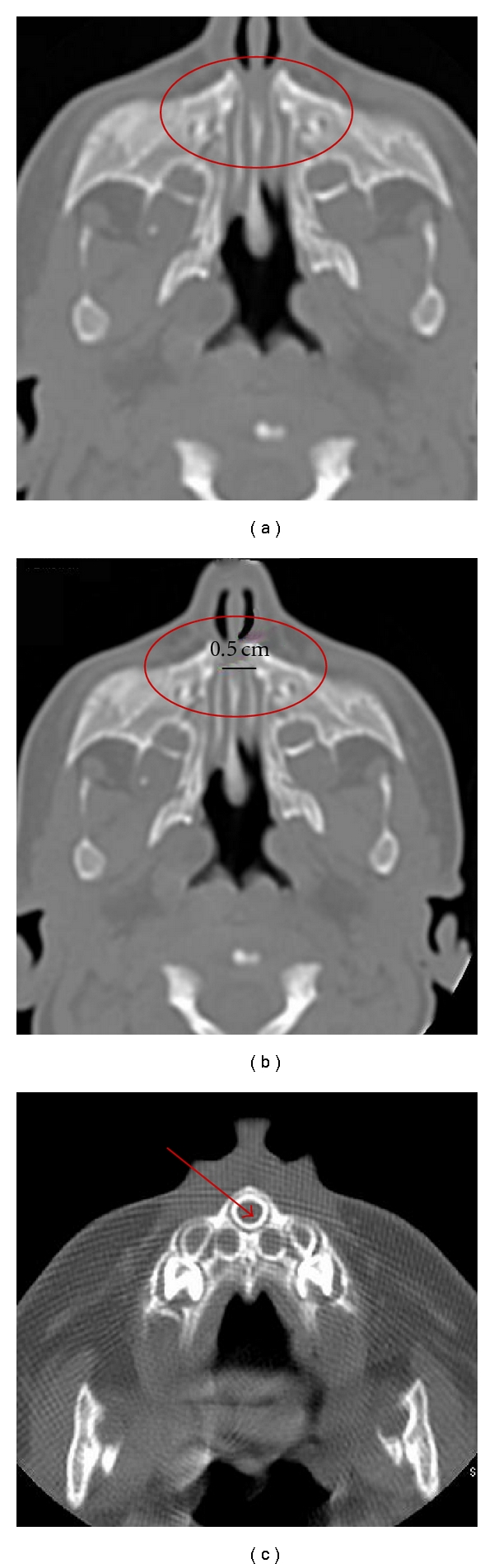
Congenital nasal pyriform aperature stenosis (a) and (b) (circle) and the patent choana. Same patient (c) with central mega incisor (arrow).

**Figure 4:**
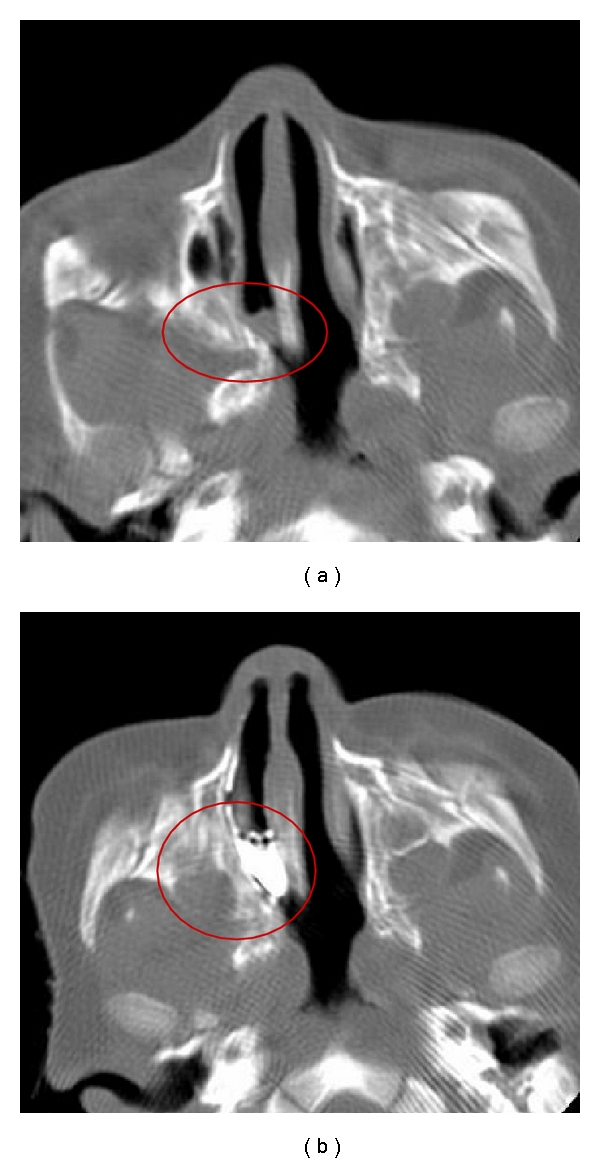
Axial CT before (a) and after (b) local contrast installation in a mixed-type patient. The membrane is thin, not as seen in plain view.

**Figure 5:**
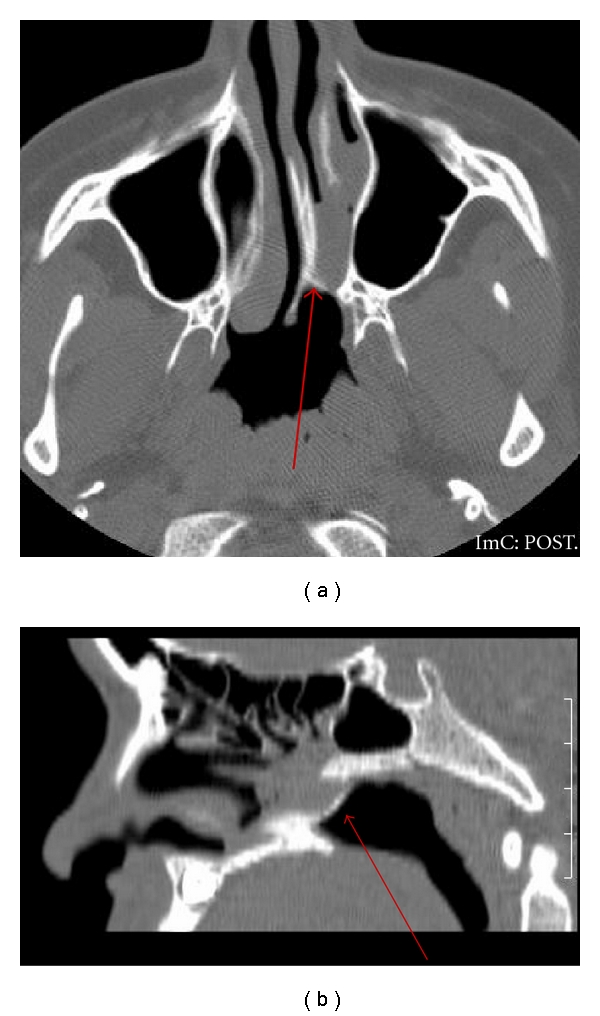
Axial (a) and sagittal reconstruction. (b) Thin bony atretic plate (arrow).

One patient (11.1%) was normal with relatively mild mucosal thickening. One patient (11.1%) had bilateral bony atresia with an ear abnormality (Figure 2). One patient (11.1%) had congenital nasal pyriform aperture stenosis and a single central mega incisor (Figure 3). Three patients (33.3%) had unilateral mixed membranous and bony atresia and local contrast delineated that thin membrane (Figure 4). One of these patients also had esophageal atresia and a tracheoesophageal fistula. Three patients (33.3%) had unilateral pure bony atresia (Figure 5). 

Of the 9 patients included in our study, the patients with the single central mega incisor were diagnosed with aperture stenosis, where the width of the pyriform aperture was 5 mm at the level of the inferior turbinate. No brain or pituitary abnormalities were detected in those patients. Only 1 of 7 patients (14.3%) was found to have bilateral bony atresia. One of these patients had several congenital abnormalities, including cardiac and renal deformities and a hypoplastic lateral semicircular canal. This patient died at the age of 4 months. 

Of the 6 patients diagnosed to have unilateral mixed or purely bony atresia, 1 patient had esophageal atresia and a tracheoesophageal fistula. The remaining patients had no other CHARGE syndrome lesions (CHARGE stands for cluster of characteristics that include coloboma of the eye, heart defects, atresia of the choanae, retardation of growth or development, genital or urinary abnormalities, and ear abnormalities and deafness). 

## 4. Discussion

Choanal atresia, consisting of a unilateral or bilateral bony membranous septum between the nose and the pharynx, occurs in approximately 1 in 5000 to 7000 live births [10]. Approximately 50% of children with bilateral choanal atresia have other congenital abnormalities The most commonly described association is with CHARGE syndrome [11]. The criteria for the diagnosis of CHARGE syndrome have been recently revised [12]. The major diagnostic criteria occur commonly in CHARGE syndrome, but they are relatively rare in other conditions. These are coloboma, choanal atresia, characteristic ear abnormalities, and brainstem and cranial nerve dysfunction. The minor diagnostic characteristics occur less commonly or are less specific for CHARGE: heart defects, genital hypoplasia, orofacial clefting, tracheoesophageal fistula, short stature, and developmental delay. 

The nature and extent of the lesion causing the nasal obstruction (including congenital causes) is best determined by CT [13]. 

**Figure 6:**
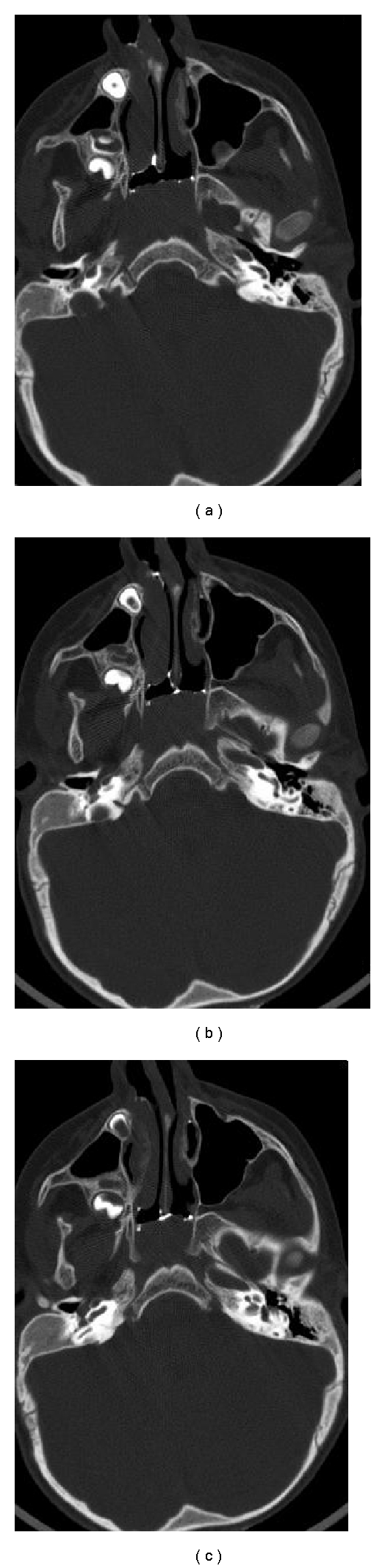
Axial CT with contrast showing hypertrophy of the inferior turbinate and patent choana.

When the diagnosis of choanal atresia is questionable, CT can differentiate other causes of bilateral nasal obstruction, such as pyriform aperture stenosis (Figure 3) or a bilateral nasolacrimal duct cyst, from choanal atresia. Unilateral causes of nasal obstruction, such as a nasal foreign body, turbinate hypertrophy (Figure 6), septal deviation, antrochoanal polyp, or nasal tumor, can also be differentiated from choanal atresia by CT. 

The main disadvantages of using CT in pediatric populations are the high radiation exposure dose and the need for sedation to avoid movement artifacts, which render the examination of fine details less reliable. Using contrast material in the nose will help to overcome that.

Over the last decade, CT has benefited from two major advances: the introduction of helical CT imaging in the early 1990s and multidetector CT acquisition in 1998 [13, 14]. The introduction of new array detector technology for multislice CT improves CT scan capabilities. This technique offers significant advantages, including improved temporal and spatial resolution, retrospective determination of slice thickness, and shorter acquisition time [15]. The isotropic effect of multislice CT combined with the ability to take different reformatting angles is again considered an advantage of this technique. 

**Figure 7:**
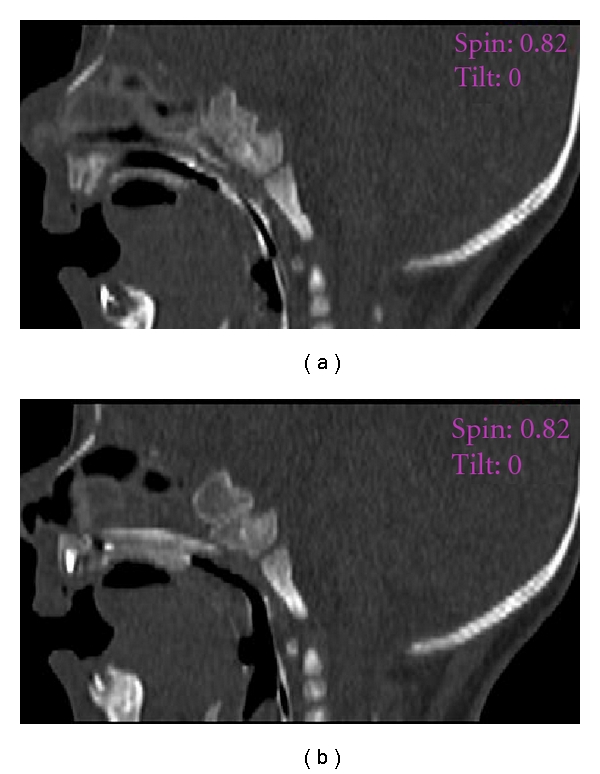
Sagital reconstruction with local contrast showing clear communication.

During the examination of patients with a questionable diagnosis of choanal atresia, it is necessary to have a scanning angle that enables us to delineate the communication of the nasal cavity with the nasopharynx. This can be achieved by changing the angle of the gantry so that the imaging plane becomes parallel to the hard palate. The difficulty of this technique is that the inadequate positioning of some patients in such an age group necessitates the repetition of the scan, resulting in a subsequent increase in the radiation dose. In addition, the angle of the imaging plane increases the risk to the child's lens. Multislice CT generates good isotropic reformatted images with a straight gantry and, hence, decreases the likelihood of repeating the scans and avoids the direct exposure of the lens. Sagittal reformatting, although not crucial, may provide a more accurate diagnosis of choanal abnormalities (Figure 7). Since reformatted scan is not as accurate as actual scan and depends upon the thickness of the scan, hence the local contrast in the nose will enhance the findings without extra radiation exposure.

In our institute, we apply low dose (50 mA) volumetric scans of the paranasal sinuses in thin axial cuts using the multislice technique with reconstruction in different planes. This low-dose technique does not affect the measurement accuracy of choanal parameters and can, therefore, be used with a subsequent decrease in the radiation dose. In postsurgical or recurrent choanal stenosis, the cause is usually a postoperative scar or an incompletely resected bony atretic plate. In such patients, we found that the local instillation of 1–3 drops of diluted nonionic contrast medium is helpful for the more accurate delineation of the communication of the posterior choanae with the nasopharynx. In some patients, even with good suction and the administration of a topical nasal vasoconstrictor, the characterization of the nature and thickness of the atretic membrane may be not accurate. The application of a local contrast agent helped to avoid such pitfalls, providing a more accurate delineation of the abnormality. This instillation did not affect the measurement of the thickness of the vomer or the width of the posterior choanae. 

In our study, one patient was normal with no clear evidence of choanal atresia or other causes that may lead to nasal obstruction, for example, turbinate hypertrophy; however, the clinical diagnosis of choanal atresia was most likely due to mucosal edema. Congenital nasal pyriform aperture stenosis was first described as a distinct entity in the radiology literature in 1988 [16] and first described clinically in 1989 [17]. A pyriform aperture width of less than 11 mm in a term infant, as measured by CT, is considered to be diagnostic of this condition [18]. As an otolaryngologist cleansing the nose and vasoconstriction will help to delineate the pathology. Local contrast is an extra tool that can enhance this information especially the thickness of the atretic plate and the condition of the mucosa as well as the vomer thickness. The later are important information to plan for surgery.

## 5. Conclusions

- Multislice CT can clearly delineate the posterior choanae and their communication with the nasopharynx.
- The application of a low-dose technique using 50–60 mA can avoid a high radiation dose to the child without any effect on the diagnostic accuracy.
- Local instillation of a contrast medium helps in postsurgical cases. It could be routinely used, even in patients without previous surgery, for a more accurate delineation of the cause of the nasal obstruction.

## Figures and Tables

**Table 1:** Patient demographics.

Group	No. of Patients	Percentage	Male : Female	Other Congenital Abnormalities	
				Number	Percentage
Normal	1	11.1%	Male		
Bilateral bony atresia	1	11.1%	1 : 1	1	100%
Congenital nasal pyriform aperture stenosis	1	11.1%	Male	1	100%
Unilateral mixed bony and membranous	3	33.3%	2 : 1	1	33.3%
Unilateral pure bony	3	33.3%	1 : 2		
